# Knowledge translation tools for parents on child health topics: a scoping review

**DOI:** 10.1186/s12913-017-2632-2

**Published:** 2017-09-29

**Authors:** Lauren Albrecht, Shannon D. Scott, Lisa Hartling

**Affiliations:** 1 Department of Pediatrics & Research Coordinator, Faculty of Nursing, University of Alberta, 5-147 Edmonton Clinic Health Academy, 11405 87 Avenue, Edmonton, AB T6G 1C9 Canada; 2 Faculty of Nursing, University of Alberta, 3rd floor Edmonton Clinic Health Academy, 11405 87 Avenue, Edmonton, AB T6G 1C9 Canada; 3 Department of Pediatrics, University of Alberta, 4-472 Edmonton Clinic Health Academy, 11405 87 Avenue, Edmonton, AB T6G 1C9 Canada

**Keywords:** Knowledge translation, Health consumers, Parents, Caregivers, Child health, Pediatrics

## Abstract

**Background:**

An emerging field of knowledge translation (KT) research has begun to focus on health consumers, particularly in child health. KT tools provide health consumers with research knowledge to inform health decision-making and may foster ‘effective consumers’. Thus, the purpose of this scoping review was to describe the state of the field of previously published effectiveness research on child health-related KT tools for parents/caregivers to understand the evidence-base, identify gaps, and guide future research efforts.

**Methods:**

A health research librarian developed and implemented search strategies in 8 databases. One reviewer conducted screening using pre-determined criteria. A second reviewer verified 10% of screening decisions. Data extraction was performed by one reviewer. A descriptive analysis was conducted and included patient-important outcome classification, WIDER Recommendation checklist, and methodological quality assessment.

**Results:**

Seven thousand nine hundred fifty two independent titles and abstracts were reviewed, 2267 full-text studies were retrieved and reviewed, and 18 articles were included in the final data set. A variety of KT tools, including single- (*n* = 10) and multi-component tools (*n* = 10), were evaluated spanning acute (*n* = 4), chronic (*n* = 5) and public/population health (*n* = 9) child health topics. Study designs included: cross-sectional (n = 4), before-after (*n* = 1), controlled before-after (*n* = 2), cohort (*n* = 1), and RCTs (*n* = 10). The KT tools were evaluated via single primary outcome category (*n* = 11) and multiple primary outcome categories (*n* = 7). Two studies demonstrated significant positive effects on primary outcome categories; the remaining studies demonstrated mixed effects (*n* = 9) and no effect (*n* = 3). Overall, methodological quality was poor; studies lacked a priori protocols (*n* = 18) and sample size calculations (*n* = 13). Overall, intervention reporting was also poor; KT tools lacked description of theoretical underpinnings (*n* = 14), end-user engagement (*n* = 13), and preliminary research (*n* = 9) to inform the current effectiveness evaluation.

**Conclusions:**

A number of child health-related knowledge translation tools have been developed for parents/caregivers. However, numerous outcomes were used to assess impact and there is limited evidence demonstrating their effectiveness. Moreover, the methodological rigor and reporting of effectiveness studies is limited. Careful tool development involving end-users and preliminary research, including usability testing and mixed methods, prior to large-scale studies may be important to advance the science of KT for health consumers.

**Electronic supplementary material:**

The online version of this article (10.1186/s12913-017-2632-2) contains supplementary material, which is available to authorized users.

## Background

It is well established that the creation of new knowledge through biomedical and health services research does not automatically lead to widespread implementation or health impacts [1]. To maximize health system resources and improve patient outcomes, it is increasingly important to close the research-practice gap by ensuring that research knowledge translates into action – a process called knowledge translation (KT). KT is defined as the synthesis, exchange, and application of knowledge to improve the health of individuals, provide more effective health services and products, and strengthen health care systems [1]. Current approaches to KT are largely focused on aligning the behaviours of health professionals with best research evidence; however, ever-increasing healthcare complexity and health professional time constraints are barriers to effective research use [2, 3]. An emerging approach to KT is directing information to health consumers (i.e., patients, parents, caregivers) to increase their knowledge and participation in health decision-making.

In the field of child health, connecting parents and caregivers to research evidence has the power to improve health decision-making and reduce health system costs [4]. Traditional approaches used by health providers to share information with parents and caregivers have been found lacking. For instance, verbal information is often brief [5] and written information is often too complex for most adults to comprehend [6, 7]. There is little guidance on the most effective approach, content, duration, and intensity of information provision for the diverse population that parents represent [8–10].

While KT interventions encompass a wide array of strategies to bridge the research-practice gap, including individual, organizational, and structural interventions [11], KT tools are a sub-group of KT interventions that present research-based information in user-friendly language and formats to provide explicit recommendations, and/or meet knowledge/information needs [12]. KT tools are particularly suited for lay audiences, including parents and caregivers. It is hypothesized that KT tools may foster and empower ‘effective consumers’ with research knowledge to inform their health decision-making [13].

The purpose of this scoping review was to identify previously published effectiveness research on child health-related KT tools for parents/caregivers. We sought to understand the breadth of KT tools that have been developed and evaluated (including their intended purpose), how they are being evaluated (including the outcomes selected), and whether they are demonstrating hypothesized effects. Understanding the evidence-base for KT tools for parents/caregivers in child health and identifying gaps in this emerging field is a critical next step to inform KT science for health consumers.

## Methods

This scoping review was guided by the rigorous, systematic methods outlined by Arksey & O’Malley [14].

### Search strategy

A comprehensive literature search was designed and implemented by a health research librarian (TC) in eight databases: Medline, Medline In-Process & Other None-Indexed Citations, EBM Reviews, Embase, PsychINFO, CINAHL, SocINDEX, and Web of science. The search included language (English only) and date restrictions (2005 – June 2015) (search strategies and terms in Additional file 1). Date restrictions reflect the advent of KT science [12, 15] and the emergence of KT targeting health consumers [16].

### Study inclusion criteria

The inclusion criteria are outlined in Table 1. In brief, we were interested in any primary research evaluating the effectiveness of a KT tool on a child health topic and targeting parents/lay caregivers. A KT tool was defined as a tangible, on-demand product presenting research-based information in user-friendly language and format(s) to provide explicit recommendations, and/or meet knowledge/information needs.

**Table 1:** Study inclusion criteria

Inclusion Criteria	Definitions & Notes
1. Primary research study	Inclusive of all study designs.
2. Evaluated effectiveness of an intervention	Defined as determining efficacy and/or effectiveness (i.e., does it work?). Studies examining functionality, feasibility, and/or acceptability to inform intervention development were excluded.
3. Intervention evaluated was a KT tool	Defined as tangible (i.e., either material or electronic) products presenting research-based information in user-friendly language and format(s) to provide explicit recommendations, and/or meet knowledge/information needs. The KT tool must be available on-demand so that the target audience can mediate its use (i.e., when to use them, how often to use them, etc.).
4. Intervention targeted parents/caregivers	Defined as individuals responsible for the health and wellbeing of child(ren) and are active-participants in child health decision making.
5. Intervention provided research-based information on child health topics	Inclusive of all child health topics.

### Study selection

One reviewer (LA) conducted primary and secondary screening using pre-determined criteria (Additional file 2). A second, independent reviewer (XW) screened 10% of all studies to verify inclusion/exclusion decisions. Interrater agreement was determined to be ‘very good’ with a kappa statistic of 0.803 [17].

### Data collection

Data were extracted by one reviewer (LA). The following general variables were extracted: authors, year of publication, country, and journal of publication. Methodological elements were also extracted, including: study design, study focus (i.e., purpose), availability of a priori protocol, study population, sample size calculation, recruitment and retention, intervention and comparison groups, data collection methods, primary outcome(s) and measures. We also extracted the results for the primary outcomes, and author conclusions. Additional variables specific to the KT tools were extracted, including: child health topic, purpose of tool, description of tool, tool development approaches (e.g., including end-users, theoretical basis, and preliminary research conducted prior to effectiveness evaluation), type of tool, and number of interacting tool elements.

### Methodological quality assessment

Scoping reviews do not typically include critical appraisal of individual studies [14, 18]. This has been acknowledged as a limitation of the Arksey & O’Malley method [19]. New methodological recommendations include methodological quality assessment to demonstrate gaps in the evidence-base and demonstrate feasibility of future systematic reviews [19]. However, studies should not to be excluded based on these methodological quality ratings [19], which is how we proceeded in this review.

For randomized controlled trials (RCTs), methodological quality was assessed by one reviewer (LA) using the Cochrane Risk of Bias Tool [20]. This tool has been deemed the most comprehensive for assessing potential for bias in RCTs [21] and has become the standard approach for systematic reviews [22]. A global quality rating of low, high or unclear risk of bias is assigned to each RCT based on seven components: random sequence generation, allocation concealment, blinding of participants and personnel, blinding of outcome assessment, incomplete outcome data, selective reporting, ‘other’ sources of bias.

For all other quantitative study designs, methodological quality was assessed by one reviewer (LA) using the Quality Assessment Tool for Quantitative Studies [23]. Content validity, construct validity, and inter-rater reliability have been established for this tool [24]. A global quality rating of weak, moderate or strong is assigned to each study based on eight components: selection bias, study design, confounders, blinding, data collection methods, withdrawals and dropouts, intervention integrity, and analysis.

### Data analysis

A descriptive analysis of the extracted variables was conducted. The WIDER Recommendations Checklist was applied to describe the reporting quality of the KT tools [25] (Additional file 3). Since the studies assessed primary outcomes at different levels, a classification scheme of outcomes for assessing patient-focused interventions was applied [26] (Additional file 4). Study results were described as positive effect, mixed effects, no effect, or unclear in relation to the intended impact on the primary outcome(s). A narrative summary of these effects was performed considering the nature of the intervention, topic, and study design features.

## Results

**Fig. 1:**
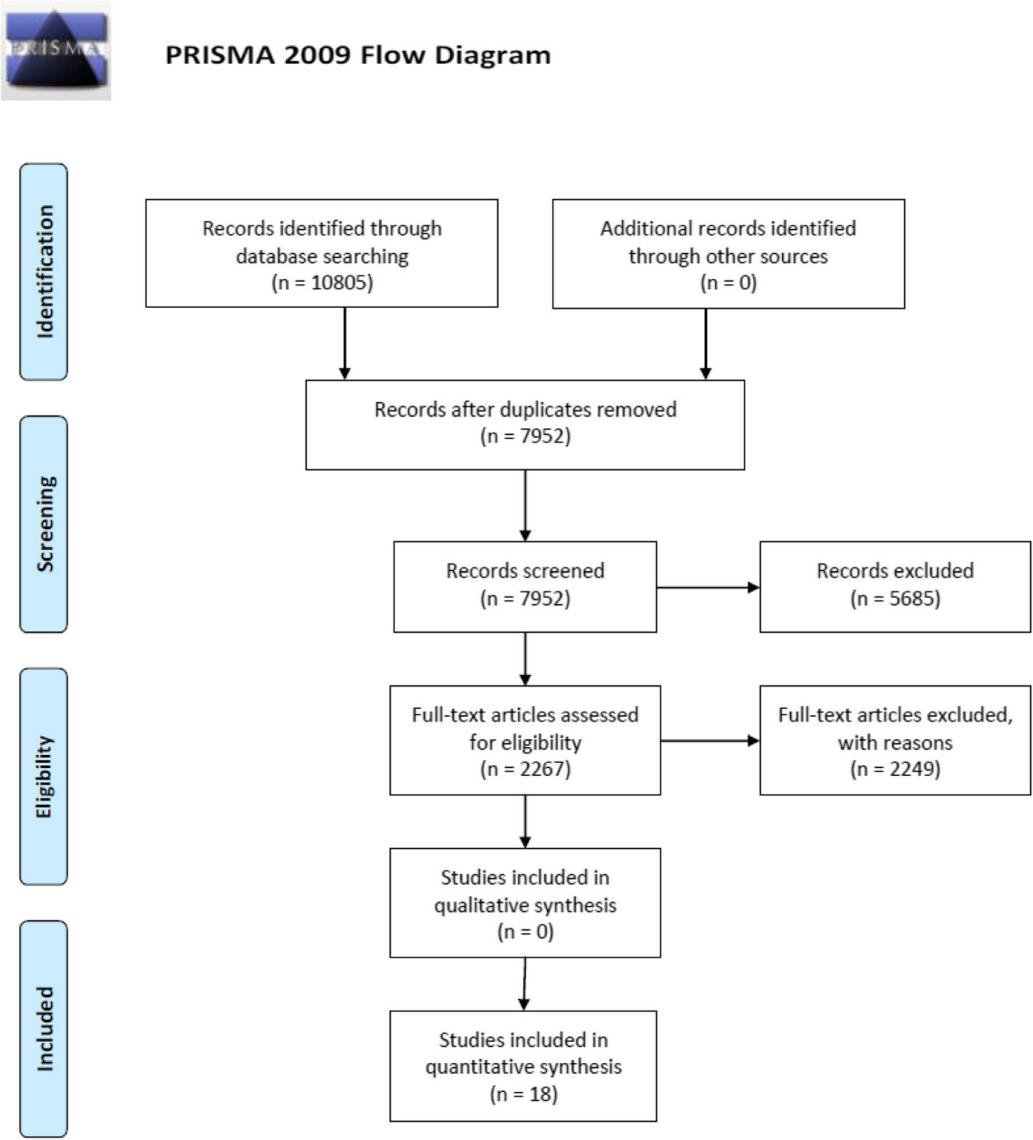
PRISMA Flow diagram. Legend: Number of studies identified by search strategy, number of studies excluded and included during primary and secondary screening, and final number of studies included

After removing duplicates, 7952 titles and abstracts were reviewed in primary screening, 2267 full-text studies were reviewed in secondary screening, and 18 studies met our inclusion criteria (Fig. 1) [27–44]. The included studies are summarized in an additional file (Additional file 5).

### KT tool interventions

The KT tools provided evidence-based information on different acute conditions (*n* = 4; e.g., gastroenteritis, tonsillitis, procedural pain, surgery), chronic conditions (*n* = 5; e.g., inherited metabolic disorders, Type I diabetes, asthma, vision impairment), and public health/health promotion topics (*n* = 9; e.g., preventive care/minor child health issues, vaccination, antibiotics use, healthy diet & physical activity, infant feeding, smoking prevention) in child health. A variety of KT tool interventions were studied, including pamphlet (*n* = 3), information sheet (*n* = 2), cartoon book (*n* = 1), book (*n* = 1), video (*n* = 1), website (*n* = 2), video + booklet (*n* = 1), 2 videos +2 booklets (*n* = 2), video + book (*n* = 1), video + pamphlet (*n* = 1), 2 videos +2 pamphlets (*n* = 1), 5 activity guides + tip sheets + newsletters (*n* = 1), and 6 books (*n* = 1). Additionally, 6 studies had KT tools as comparison/control conditions; these tools included, pamphlet [42], 1 video +1 pamphlet [34, 35], 2 pamphlets [33], 2 information sheets [44] 5 pamphlets [32].

Another approach to classify KT tools is to examine the number of different components (i.e., single or multiple) within the intervention (as shown in the above list) (Table 2). In nine studies, KT tools featured one (single) stand-alone component (e.g., information sheet) [27, 29, 30, 36, 38–42]; two single-component KT tools were compared and evaluated in one of these studies [42]. In nine studies, KT tools included multiple (more than one) components that worked in tandem (e.g., pamphlet + video) [28, 31–35, 37, 43, 44]; two multi-component KT tools were compared and evaluated in five of these studies [32–35, 44].

The quality of reporting of the KT tools was described using the WIDER Recommendations Checklist [25] (Table 2). Overall reporting quality was low. For the ‘Detailed Description of Intervention’ recommendation there were 8 components; included studies achieved between 3 and 6 components with a mean of 4 and a mode of 5 components. Generally included studies did not report on: characteristics of those delivering the intervention, the intensity of the intervention, and adherence/fidelity to delivery protocols. For the ‘Clarification of Assumed Change Process and Design Principles’ recommendation there were 3 components. Change techniques used in the intervention were the most reported component (12/18 studies). Causal processes targeted by change techniques and intervention development processes were rarely reported (5/18 studies for both components). Four of 18 studies satisfied the third recommendation, ‘Access to Intervention Manuals/Protocols’. For the fourth recommendation, ‘Detailed Description of Active Control Conditions’, a variety of control conditions were present (i.e., active control, no active control, multiple control groups, no control group).

### Study designs

Five different quantitative study designs were represented: cross-sectional (*n* = 4) [30, 31, 36, 40], before-after (*n* = 1) [39], controlled before-after (*n* = 2) [38, 41], cohort (*n* = 1) [33], and RCT (*n* = 10) [27–29, 32, 34, 35, 37, 42–44]. No qualitative studies met the inclusion criteria.

### Methodological quality

Ten RCTs were assessed for risk of bias (Table 3). Five studies were assessed as high risk of bias [27–29, 32, 44]; the most frequent reason for high risk of bias was lack of blinding of participants and personnel. Five studies were determined to have unclear risk of bias [34, 35, 37, 42, 43]; the most frequent reason for unclear risk of bias was the possibility of selective outcome reporting. None of the included studies were assessed as low risk of bias overall.

All eight of the quantitative, non-RCT studies had a global methodological quality rating of weak [30, 31, 33, 38–41] (Table 4). The most problematic domains across studies were ‘study design’ and ‘data collection and methods’; all studies were weak with respect to these domains except one.

### Primary outcomes

Primary outcomes were classified into four categories using the Outcomes of Interest for Assessing Patient-Focused Interventions classification scheme [26] (Table 5). It was possible for one outcome category to encompass several different outcome measures (e.g., self-efficacy measures and perceived barrier measures are both captured under the Patients’ Experience outcome category) (Additional file 4). Overall, 11 studies assessed one primary outcome category to determine the effectiveness of KT tools: patients’ knowledge (*n* = 3) [37, 41, 44]; patients’ experience (*n* = 1) [30]; health behaviour and health status (*n* = 7) [29,31,3234,35,40,42]. None of the included studies assessed outcomes in the health services utilization and cost category of the outcome classification scheme.

**Table 2:** WIDER Recommendations Checklist for Intervention Reporting Quality

Author (Year)	Detailed Description of Intervention (Y/N)								Clarification of Assumed Change Process and Design Principles (Y/N)			Access to Intervention Manuals/ Protocols	Detailed Description of Active Control Conditions (Y/N)			
	1	2	3	4	5	6	7	8	1	2	3	Y/N	1	2	3	4
Bailey (2015)	N	Y	Y	Y	Y	N	N	N	N	N	N	N			Y	
Bauchner (2001)	N	Y	Y	Y	N	Y	N	Y	Y	Y	N	N			Y	
Christakis (2006)	N	Y	Y	N	N	N	N	Y	N	Y	N	N	Y		Y	
Dempsey (2006)	N	Y	Y	Y	N	N	Y	Y	N	Y	Y	Y			Y	
Evans (2009)	N	Y	Y	Y	N	Y	N	Y	N	Y	N	N				Y
Jackson (2006)	N	Y	Y	Y	N	N	N	N	N	N	N	N	Y			
	N	Y	Y	Y	N	N	N	N	N	N	N	N	control condition			
Nordfeldt (2002)	N	Y	Y	Y	N	Y	N	Y	Y	Y	Y	N	Y			
	N	Y	Y	Y	N	Y	N	Y	Y	Y	Y	N	Y			
Nordfeldt (2003)	N	Y	Y	Y	N	Y	N	Y	Y	Y	Y	N	Y			
	N	Y	Y	Y	N	Y	N	Y	Y	Y	Y	N	Y			
Nordfeldt (2005)	N	Y	Y	Y	N	Y	N	Y	Y	Y	Y	N	Y			
	N	Y	Y	Y	N	Y	N	Y	Y	Y	Y	N	Y			
Ranjit (2015)	N	Y	Y	Y	N	N	N	Y	N	N	Y	N			Y	
Reich (2010)	N	Y	Y	Y	Y	Y	N	Y	N	N	N	N	Y		Y	
Scheinmann (2009)	N	Y	Y	Y	N	N	N	N	N	N	N	N			Y	
Skranes (2015)	N	Y	Y	Y	N	N	N	N	N	Y	N	Y			Y	
Sustersic (2012)	N	Y	Y	Y	N	Y	N	N	N	Y	N	Y			Y	
Taddio (2014)	N	Y	Y	Y	N	Y	N	Y	N	Y	N	Y			Y	
Tjiam (2012)	N	Y	Y	Y	N	N	N	N	N	N	N	N	Y	Y		
	N	Y	Y	Y	N	N	N	N	N	N	N	N	Y	Y		
Wakimizu (2009)	N	Y	Y	Y	N	Y	N	Y	Y	Y	N	N	Y			
Wilson (2006)	N	Y	Y	Y	N	N	N	N	N	Y	N	N	Y			
	N	Y	Y	Y	N	N	N	N	N	Y	N	N	control condition

**Table 3:** Risk of bias assessment of included RCTs

Author (year)	Sequence generation	Allocation concealment	Blinding participants & personnel	Blinding of outcome assessment	Incomplete outcome data	Selective outcome reporting	Other sources of bias	Overall score
Bailey (2015)	Low	Low	Unclear	Unclear	Low	High	High	High
Bauchner (2001)	Unclear	Unclear	High	Unclear	Low	Unclear	High	High
Christakis (2006)	Low	Low	High	Low	Unclear	Unclear	Low	High
Jackson (2006)	Unclear	Unclear	High	Low	Low	Unclear	Low	High
Nordfeldt (2003)	Low	Low	Low	Low	Unclear	Unclear	Low	Unclear
Nordfeldt (2005)	Low	Low	Low	Low	Unclear	Unclear	Low	Unclear
Reich (2010)	Unclear	Unclear	Unclear	Unclear	Unclear	Unclear	Low	Unclear
Tijam (2013)	Unclear	Unclear	Unclear	Low	Low	Unclear	Low	Unclear
Wakimizu (2009)	Low	Unclear	Low	Low	Low	Unclear	Low	Unclear
Wilson (2006)	Unclear	Unclear	Unclear	Unclear	High	Unclear	Unclear	High

**Table 4:** Quality assessment of included non-RCT studies

Author (year)	Selection bias	Study design	Confounders	Blinding	Data collection methods	Withdrawals & drop-outs	Global rating
Dempsey (2006)	Weak	Weak	Strong	Moderate	Weak	Moderate	Weak
Evans (2009)	Moderate	Weak	Weak	Moderate	Weak	Strong	Weak
Nordfeldt (2002)	Moderate	Moderate	Weak	Moderate	Weak	Weak	Weak
Ranjit (2015)	Weak	Weak	Weak	Moderate	Weak	Weak	Weak
Scheinman (2010)	Weak	Weak	Strong	Moderate	Weak	Moderate	Weak
Skranes (2015)	Weak	Weak	Weak	Moderate	Weak	Moderate	Weak
Sustersic (2013)	Moderate	Weak	Strong	Moderate	Weak	Strong	Weak
Taddio (2014)	Weak	Weak	Strong	Moderate	Strong	Strong	Weak

**Table 5:** Effectiveness of KT tools on primary outcome categories

Study design	First author (year)	Single/Multiple component KT tool groups (specific KT Tool)	Health category (topic)	Primary Outcome Categories		
				Patient’s knowledge	Patient’s experience	Health behaviour & health status
Cross-sectional	Dempsey (2006)	Single component (information sheet)	Public health (Vaccination)	–	no effect	–
	Evans (2009)	Multi-component (video + book)	Chronic (Inherited metabolic disorders)	–	–	unclear
	Ranjit (2015)	Single component (book)	Public health (Healthy diet & physical activity)	no effect	mixed effects	no effect
	Sustersic (2013)	Single component (pamphlet)	Acute (Gastroenteritis, tonsillitis)	–	–	mixed effects
Before-after	Skranes (2015)	Single component (website)	Public health (Minor child health conditions)	positive effect	no effect	**–**
Controlled before-after	Scheinman (2010)	Single component (video)	Public health (Infant feeding)	mixed effects	–	mixed effects
	Taddio (2014)	Single component (pamphlet)	Acute (Procedural pain management)	no effect	–	–
Cohort	Nordfeldt (2002)	Multi-component (2 videos +2 pamphlets)	Chronic (Type I diabetes)	–	unclear	unclear
		Multi-component (2 pamphlets)				
RCT	Bailey (2015)	Single component (information sheet)	Acute (Surgical pain management)	positive effect	positive effect	mixed effects
	Bauchner (2001)	Multi-component (video + pamphlet)	Public health (Antibiotics use)	no effect	no effect	mixed effects
	Christakis (2006)	Single component (tailored website)	Public health (Preventive care)	–	–	mixed effects
	Jackson (2006)	Multi-component (5 printed activity guides + series of tip sheets for parents + series of newsletters for children)	Public health (Smoking prevention)	–	–	positive effect
		Multi-component (5 information sheets)				
	Nordfeldt (2003)	Multi-component (2 videos +2 booklets)	Chronic (Type I diabetes)	–	–	mixed effect
		(1 video +1 booklet)				
	Nordfeldt (2005)	Multi-component (2 videos +2 booklets)	Chronic (Type I diabetes)	–	–	positive effect
		Multi-component (1 video +1 booklet)				
	Reich (2010)	Multi-component (6 books)	Public health (Minor child health conditions)	mixed effects	–	–
	Tijam (2013)	Single component (cartoon book)	Chronic (Vision impairment)	–	–	mixed effects
		Single component (pamphlet)				
	Wakimizu (2009)	Multi-component (video + booklet)	Acute (Surgery)	mixed effects	mixed effects	–
	Wilson (2006)	Multi-component (2 pamphlets)	Public health (Vaccination)	no effect	–	–
		Multi-component (2 information sheets)

**Table 6:** Additional key study design & intervention development features

Study Design	Author (year)	A priori protocol publicly available (Y/N)	Sample size calculation provided (Y/N)	Sufficiently powered for primary outcome (Y/N/?)	Theory-based intervention (Y/N/?)	End-users involved in intervention development (Y/N/?)	Preliminary qualitative/ feasibility/ pilot work referenced (Y/N)
Cross- sectional	Dempsey (2006)	N	Y	Y	Y	?	N
	Evans (2009)	N	N	?	N	Y	N
	Ranjit (2015)	N	N	?	Y	N	N
	Sustersic (2013)	N^a^	Y	Y	?	?	Y
Before-after	Skranes (2015)	N	N	?	N	N	N
Controlled before-after	Scheinman (2010)	N	N	?	N	Y	Y
	Taddio (2014)	N	Y	N	N	?	Y
Cohort	Nordfeldt (2002)	N	N	?	Y	Y	Y
RCT	Bailey (2015)	N^a^	N	?	?	?	N
	Bauchner (2001)	N	Y	Y	N	Y	Y
	Christakis (2006)	N^a^	Y	N	N	?	N
	Jackson (2006)	N	N	?	?	?	Y
	Nordfeldt (2003)	N	N	?	N	?	Y
	Nordfeldt (2005)	N	N	?	N	?	Y
	Reich (2010)	N^a^	N	?	N	?	N
	Tjiam (2013)	N	N	?	N	N	Y
	Wakimizu (2009)	N	N	?	Y	Y	N
	Wilson (2006)	N	N	?	N	?	N

^a^Retrospective protocol registration

Seven studies assessed KT tool effectiveness with multiple primary outcome categories. Four of these studies identified and assessed primary outcomes in two different outcome categories: patients’ knowledge and patients’ experience (*n* = 2) [39, 43]; patients’ knowledge and health behaviour/health status (*n* = 1) [38]; and patients’ experience and health behaviour/health status (*n* = 1) [33]. Three studies identified primary outcomes in three different outcome categories: patients’ knowledge, patients’ experience, and health behaviour/health status categories [27, 28, 36].

### Study results

A summary of study results is presented in Additional file 4. Of the 18 included studies, two studies demonstrated significant positive effects on the primary outcome [32, 35]. Both studies were RCTs that assessed the effectiveness of multi-component KT tools using the health behaviour/health status primary outcome category. In both studies the primary outcome was assessed using one single measure at two time points (i.e., baseline and follow-up) with a long follow up period (i.e., 2 years, 3 years). Jackson et al. (2006) compared 2 multi-component KT tools, with the more dynamic tool (i.e., 5 printed activity guides with supplementary fact sheets for parents and newsletters for children vs 5 pamphlets) demonstrating effectiveness in delaying initiation of smoking. This study was assessed to have high risk of bias due to lack of blinding of study participants and personnel (Table 3). Nordfeldt et al. (2005) compared 2 multi-component KT tools (i.e., 2 videos +2 booklets vs 1 video +1 booklet with different information for each study arm) and a usual care control group, with the more dynamic and specific tool (2 videos +2 booklets on self-control and treatment information vs 1 video +1 booklet on general diabetes information) demonstrating effectiveness on reducing yearly incidence of severe hypoglycemia needing assistance. This study was assessed to have unclear risk of bias with respect to incomplete outcome data and selective outcome reporting (Table 3).

Two additional studies demonstrated significant positive effects on at least one of the identified primary outcome categories [27, 39]. Both studies assessed the effectiveness of single-component KT tools. Skranes et al. (2015) utilized a before-after design and determined that a website was effective for improving mothers’ knowledge of minor child health conditions, but not mothers’ experience (i.e., self-perceived anxiety) over a six to 12-month follow-up period. The methodological quality was assessed as weak (Table 4). Bailey et al. (2015) conducted a RCT and determined that an information sheet was effective for improving knowledge and experience with respect to tonsillectomy surgery pain management, but not health behaviour/status over a 10-day follow-up period. This study was assessed to have a high risk of bias due to selective outcome reporting and other sources of bias (i.e., baseline imbalances in study groups) (Table 3).

Nine studies demonstrated a combination of mixed effects and no effect on primary outcome categories (both single and multiple primary outcome categories) [28, 29, 34, 36–38, 40, 42, 43]. These studies were diverse in terms of the KT tool interventions (i.e., a variety of single and multi-component KT tools), study designs, and number of outcomes within and between the three primary outcome categories. Four of nine studies had longer follow-up periods (i.e., from 10 weeks to three years), three had shorter follow-up periods (i.e., less than 8 weeks), and two had undefined follow-up periods (i.e., referred to as post-intervention assessment with no timing provided).

Three studies showed no effect on the primary outcome categories [30, 41, 44]. These studies were conducted using different designs: cross-sectional [30], controlled before-after [41], and RCT [44]. They represented both single and multi-component KT tools; however, all three KT tools were non-electronic, written materials (i.e., information sheet, pamphlets). The three studies measured single, proximal outcome categories (i.e., knowledge, experience) over a short follow-up period (i.e., 2 weeks, 2 months). The methodological quality of two studies was assessed as weak [30, 41] (Table 4) and the third study was determined to have high risk of bias [44] (Table 3).

### Additional key study features

To contextualize the study results and methodological quality ratings, additional data were extracted about key study features with a focus on methodological and intervention development variables (Table 6). References to trial registration and a priori protocols were extracted and relevant databases were searched. None of the 18 studies had an a priori protocol publicly available; however, four studies were registered retrospectively [27, 29, 37, 40]. Five of 18 studies provided a sample size calculation [28–30, 40, 41] and of those studies, three were sufficiently powered to detect the desired change in the primary outcome [28, 30, 40]. Four of 18 studies described a theoretical basis for the KT tool intervention [30, 33, 36, 43] and five of 18 studies explicitly described end-user involvement in intervention development [28, 31, 33, 38, 43]. Finally, nine studies described or referenced preliminary research (i.e., qualitative, feasibility, pilot studies) that informed the current KT tool effectiveness study [28, 32–35, 38, 40, 41]. There were no discernable patterns between these variables and the effectiveness of the KT tools.

## Discussion

This scoping review has demonstrated that several different KT tools have been specifically designed for parents/caregivers on diverse child health topics, which include a variety of single- and multi-component strategies. Few KT tools demonstrated positive effects for primary outcomes; the majority of studies showed mixed effects within and between primary outcome categories. Only two studies showed strictly positive effects and both evaluated multi-component KT tools. Three studies showed no effect and these evaluated single-component KT tools, specifically they were all non-electronic, written materials. This suggests that multi-component KT tools may be more effective for health consumers, specifically parents and caregivers. While we did not conduct formal comparisons, these findings contradict previous research indicating the effectiveness of patient-focused interventions decreases as the number of intervention components number increases [45].

This review demonstrated that the most common design was the RCT (*n* = 10), which is recognized as the most rigorous design for evaluating effectiveness [46]. All included RCTs were assessed as unclear or high risk of bias; further, included non-RCT studies all had substantial methodological weaknesses. Given these methodological weaknesses, it might be expected that interventions would be more likely to demonstrate an effect, particularly for the primary outcomes; however, most studies did not demonstrate significantly positive results on the primary outcomes of interest. This raises three considerations: 1) was the design or certain design features (e.g., sample size, nature of the comparison) inappropriate or inadequate to assess effectiveness; 2) were appropriate outcomes selected and measured to accurately assess intended impact and establish effectiveness; or 3) have the KT tools been appropriately developed and incrementally assessed to establish effectiveness?

There were several design/methodological issues that may have impacted the effectiveness results. None of the included studies was shown to have low risk of bias (i.e., high or unclear risk of bias ratings only). Low methodological quality ratings were due to deficiencies in multiple categories in both tools. Interestingly, no a priori protocols were available and only 4 studies retrospectively registered their protocols. While a priori protocols may not yet be standard for all study designs, it is standard practice to register a priori protocols for RCTs [47, 48]. Additionally, only 3 studies (16.7%) demonstrated adequate power to detect statistical significance of the primary outcomes. This information, generally provided in study protocols (if not also in primary publications), is a key aspect of effective comparative studies in health research [49]. With strict journal length restrictions, it is difficult to determine if high risk of bias/weak methodological quality can be attributed to lack of reporting and/or poor study design and execution; however, the publication of study protocols has been proposed as an important approach in the primary prevention of poor medical/health research [50], particularly selective outcome reporting [51].

There is little agreement on the best outcomes and measures to determine effectiveness of patient-focused interventions [26]. Applying the Coulter & Ellins patient-important outcomes framework was useful because it helped to reduce the ‘noise’ and classify multiple outcome measures within and across four distinct outcome categories. Studies used many different outcome measures across a variety of proximal (i.e., patients’ knowledge) and distal (i.e., health services utilization/health behavior) outcome categories. Just under half (44%) of studies skipped proximal outcomes (i.e., knowledge) and instead only measured more distal, behaviour-related outcomes. Two such studies showed statistically significant positive effects of the KT tools, but methodological quality concerns (i.e., high and unclear ratings) and no a priori protocol or sample size estimation limit our confidence in the link between the KT tool and these distal, health behaviour and health status changes. There may be other mitigating factors in the ‘black box’ between the interventions and outcomes. Additionally, the use of multiple outcome measures within the same primary outcome category and/or measuring multiple primary outcome categories most often resulted in mixed effectiveness (*n* = 9). It is difficult to interpret these results without authors’ providing explicit rationale linking primary outcome(s) and measures to the intended effect of the KT tools. It is important to note that only four studies described the theoretical basis for the KT tool; more explicit theoretical underpinnings may help in tool development and linking tools to intended outcomes.

Unfortunately, the overall poor intervention reporting quality in the literature [52–55], including development approaches/methods [46], theoretical basis [56, 57], and end-user involvement [58], results in limited understanding of intervention components and relationship/interaction between these components, which are responsible for observed changed and desired effects on outcomes [59]. These issues were exemplified in this review with poor reporting across all WIDER Recommendation categories. Without detailed understanding of these important elements, KT tool sustainability, replication, scale-up and future development efforts are limited [25].

Effectiveness evaluation is typically resource intensive, yet we need to understand whether KT tools are effective for a lay audience prior to large-scale implementation. Formative research (i.e., qualitative, feasibility, pilot studies) prior to launching into effectiveness evaluation may be essential to attend to intervention development and implementation issues, refine effectiveness evaluation protocols, including most appropriate outcomes and measures, and ensure potential impact [57, 60]. However, only half (*n* = 9) of the included studies described or referenced preliminary research that was conducted to inform the current effectiveness evaluation study. Both studies in this review that demonstrated significant positive effects on primary outcomes referenced such preliminary research. Unfortunately, these studies did not provide sufficient detail to guide future KT tool evaluations; however, future research could attend to this need.

There is also a growing body of literature to support the use of qualitative research in the design and implementation of RCTs [61–64]. Qualitative research has been used to add value to trials in the areas of bias, efficiency, ethics, implementation, interpretation, relevance, success, and validity [65]. Novel study designs, beyond RCTs, possibly including mixed methods, may explain why the KT tools worked or not, help explain and interpret effectiveness results, and explore the implementation process [57].

### Strengths & limitations

This scoping review provides a detailed summary of the state of the science for the emerging field of KT tools for parents and caregivers on child health topics. By conducting critical appraisal using two rigorous frameworks for multiple study design types, this review offers important methodological advancement of the Arksey & O’Malley (2005) scoping review method [14, 18, 19]. One limitation is the lack of a second reviewer to verify data extraction and critical appraisal, as would be expected in a systematic review. Another limitation was the lack of a classification scheme for the KT tools; the Coulter & Ellins patient-focused intervention classification was not used because it had a broader scope than desired [26]. Multiple, overlapping frameworks are a persistent problem in the KT field [66, 67]; however, the recently published AIMD meta-framework may be the solution [68] and future research should explore KT tool development, reporting, and classification with this new framework.

## Conclusions

KT tools offer a promising approach to communicate complex health information to health consumers. While a breadth of KT tools have been developed to provide research-based information on a wide variety of acute, chronic and public health/health promotion topics in child health, improved reporting is essential to ensure intervention design is appropriate for desired change and that well designed interventions are replicable. Additionally, increased methodological rigor is needed to determine the effectiveness of the KT tools. This includes the publication of a priori protocols, sample size calculations, primary outcome identification, and attending to multiple outcome measures and mixed results. More preliminary research, including KT tool development involving the target end-users and usability testing prior to large-scale trials, may be important to optimize KT tool effectiveness. Further, ensuring all necessary intervention and methodological components are attended to before and during effectiveness evaluation will help provide a more solid scientific base for KT targeting health consumers.

## Additional files

Additional file 1: Search strategies. (DOCX 31 kb)

Additional file 2: Secondary screening criteria. (DOCX 17 kb)

Additional file 3: WIDER recommendations to improve reporting of the content of behavior change interventions. (DOCX 14 kb)

Additional file 4: Outcomes of interest for assessing patient-focused interventions. (DOCX 15 kb)

Additional file 5: Summary of included studies (*n*=18). (DOCX 32 kb)

## Abbreviations

KT: knowledge translation
RCT: randomized controlled trial
