# Supplementary material for: Knowledge translation tools for parents on child health topics: a scoping review
Source: BMC Health Serv Res. 2017 Sep 29;17:686. doi: 10.1186/s12913-017-2632-2 (PMC5622461; doi:10.1186/s12913-017-2632-2)
Supplement: Supplementary file 1 — Search strategies. (DOCX 31 kb) [file 12913_2017_2632_MOESM1_ESM.docx]

| **Platform** | **Database** | **Date search was run** | **Number of citations retrieved** |
| --- | --- | --- | --- |
| Ovid | EBM Reviews – Cochrane Central Register of Controlled Trials | 15 June 2015 | 292 |
|  | Embase 1988- | 15 June 2015 | 3238 |
|  | MEDLINE 1946- | 15 June 2015 | 2347 |
|  | MEDLINE In-Process & Other Non-Indexed Citations | 15 June 2015 | 212 |
|  | PsycINFO 1987- | 15 June 2015 | 2210 |
| EBSCOhost | CINAHL | 15 June 2015 | 1242 |
|  | SocINDEX | 15 June 2015 | 225 |
| Web of Science | Science Citation Index Expanded 1900-; Social Sciences Citation Index 1900-; Conference Proceedings Citation Index- Science 1990-; Conference Proceedings Citation Index- Social Science & Humanities 1990-; Book Citation Index– Science 2005-; Book Citation Index– Social Sciences & Humanities 2005- | 15 June 2015 | 1039 |
|  |  | Sub-Total | 10805 |
|  |  |  |  |

# MEDLINE 1946, In-Process & Other Non-Indexed Citations

1. patient education handout/

2. books/ or books, illustrated/ or cookbooks as topic/ or pamphlets/ or Cartoons as Topic/

3. Mass Media/

4. newspapers/ or periodicals as topic/

5. video-audio media/ or "instructional films and videos"/ or interactive tutorial/ or webcasts/

6. internet/ or blogging/ or social media/

7. Music/

8. or/2-7

9. exp Consumer Health Information/

10. 8 and 9

11. (storybook* or comic* or cartoon* or ebook* or e-book* or story or poem* or poetry or comic strip* or photonovella* or photo novella* or fotonovela* or photo diary or photo diaries or photodiary or photodiaries or flipchart* or flip chart* or storyboard* or story board* or printed health material*).tw,kf.

12. (pamphlet* or information sheet* or newsletter* or digital animation* or cartoon* or gif or infographic* or podcast* or social media or twitter or facebook or blog or blogs or face book or song or songs or youtube).tw,kf.

13. (book or books or video or videos or website or web site* or game or games or app or smart phone* or smartphone*).tw,kf.

14. or/11-13

15. ((health or consumer* or patient* or parent* or caregiver*) adj2 (information or education*)).tw,kf.

16. exp Consumer Health Information/

17. 15 or 16

18. 14 and 17

19. 1 or 10 or 18

20. exp health behavior/ or illness behavior/

21. self care/ or self medication/ or risk reduction behavior/ or disease management/ or "Medication Therapy Management"/

22. ((health or illness) adj behavio?r).tw,kf.

23. (self adj (manag* or care or administ* or monitor* or efficac* or medicat* or mainten* or treat*)).tw,kf.

24. or/20-23

25. 19 and 24

26. "diffusion of innovation"/

27. (research adj2 ("use" or utili#ation or adopt* or implement* or disseminat* or uptake or support)).tw,kf.

28. (knowledge adj2 ("use" or utili#ation or adopt* or implement* or disseminat* or uptake or transfer* or translat* or support)).tw,kf.

29. (evidence adj2 ("use" or utili#ation or adopt* or implement* or disseminat* or uptake or transfer* or translat* or support)).tw,kf.

30. (implementation adj1 (science or research or intervention)).tw,kf.

31. exp Translational Medical Research/

32. exp Organizational Innovation/

33. exp Information Dissemination/

34. exp Translations/

35. exp Evidence-Based Practice/

36. knowledge/

37. Health Knowledge, Attitudes, Practice/

38. knowledge.ti.

39. or/26-38

40. 19 and 39

41. 25 or 40

42. or/27-30

43. (game or games or app or smart phone* or smartphone*).tw,kf.

44. (book or books or video* or website or web site*).ti.

45. (book or books or video or videos or website or web site*).ab. /freq=2

46. or/11-12,43-45

47. 42 and 46

48. 41 or 47

49. limit 48 to yr="2000 -Current"

# EBM Reviews Cochrane Central Register of Controlled Trials

1. patient education handout/

2. books/ or books, illustrated/ or cookbooks as topic/ or pamphlets/ or Cartoons as Topic/

3. Mass Media/

4. newspapers/ or periodicals as topic/

5. video-audio media/ or "instructional films and videos"/ or interactive tutorial/ or webcasts/

6. internet/ or blogging/ or social media/

7. Music/

8. or/2-7

9. exp Consumer Health Information/

10. 8 and 9

11. (storybook* or comic* or cartoon* or ebook* or e-book* or story or poem* or poetry or comic strip* or photonovella* or photo novella* or fotonovela* or photo diary or photo diaries or photodiary or photodiaries or flipchart* or flip chart* or storyboard* or story board* or printed health material*).tw.

12. (pamphlet* or information sheet* or newsletter* or digital animation* or cartoon* or gif or infographic* or podcast* or social media or twitter or facebook or blog or blogs or face book or song or songs or youtube).tw.

13. (book or books or video or videos or website or web site* or game or games or app or smart phone* or smartphone*).tw.

14. or/11-13

15. ((health or consumer* or patient* or parent* or caregiver*) adj2 (information or education*)).tw.

16. exp Consumer Health Information/

17. 15 or 16

18. 14 and 17

19. 1 or 10 or 18

20. exp health behavior/ or illness behavior/

21. self care/ or self medication/ or risk reduction behavior/ or disease management/ or "Medication Therapy Management"/

22. ((health or illness) adj behavio?r).tw.

23. (self adj (manag* or care or administ* or monitor* or efficac* or medicat* or mainten* or treat*)).tw.

24. or/20-23

25. 19 and 24

26. "diffusion of innovation"/

27. (research adj2 ("use" or utili#ation or adopt* or implement* or disseminat* or uptake or support)).tw.

28. (knowledge adj2 ("use" or utili#ation or adopt* or implement* or disseminat* or uptake or transfer* or translat* or support)).tw.

29. (evidence adj2 ("use" or utili#ation or adopt* or implement* or disseminat* or uptake or transfer* or translat* or support)).tw.

30. (implementation adj1 (science or research or intervention)).tw.

31. exp Translational Medical Research/

32. exp Organizational Innovation/

33. exp Information Dissemination/

34. exp Translations/

35. exp Evidence-Based Practice/

36. knowledge/

37. Health Knowledge, Attitudes, Practice/

38. knowledge.ti.

39. or/26-38

40. 19 and 39

41. 25 or 40

42. or/27-30

43. (game or games or app or smart phone* or smartphone*).tw.

44. (book or books or video* or website or web site*).ti.

45. (book or books or video or videos or website or web site*).ab. /freq=2

46. or/11-12,43-45

47. 42 and 46

48. 41 or 47

49. limit 48 to yr="2000 -Current"

# Embase

1. book/

2. exp mass communication/

3. music/

4. or/1-2

5. consumer health information/

6. patient information/

7. 5 or 6

8. 4 and 7

9. (storybook* or comic* or cartoon* or ebook* or e-book*or story or poem* or poetry or comic strip* or photonovella* or photo novella* or fotonovela* or photo diary or photo diaries or photodiary or photodiaries or flipchart* or flip chart* or storyboard* or story board* or printed health material*).tw.

10. (pamphlet* or information sheet* or newsletter* or digital animation* or cartoon* or gif or infographic* or podcast* or social media or twitter or facebook or blog or blogs or face book or song or songs or youtube).tw.

11. (book or books or video or videos or website or web site* or game or games or app or smart phone* or smartphone*).tw.

12. or/9-11

13. ((health or consumer* or patient* or parent* or caregiver*) adj2 (information or education*)).tw.

14. consumer health information/ or patient information/

15. 13 or 14

16. 12 and 15

17. 8 or 16

18. exp health behavior/

19. illness behavior/

20. exp self care/

21. disease management/

22. ((health or illness) adj behavio?r).tw.

23. (self adj (manag* or care or administ* or monitor* or efficac* or medicat* or mainten* or treat*)).tw.

24. or/18-23

25. 17 and 24

26. (research adj2 ("use" or utili#ation or adopt* or implement* or disseminat* or uptake or support)).tw.

27. (knowledge adj2 ("use" or utili#ation or adopt* or implement* or disseminat* or uptake or transfer* or translat* or support)).tw.

28. (evidence adj2 ("use" or utili#ation or adopt* or implement* or disseminat* or uptake or transfer* or translat* or support)).tw.

29. (implementation adj1 (science or research or intervention)).tw.

30. translational research/

31. information dissemination/

32. exp evidence based practice/

33. knowledge/

34. knowledge.ti.

35. or/26-34

36. 17 and 35

37. 25 or 36

38. or/26-29

39. (game or games or app or smart phone* or smartphone*).tw.

40. (book or books or video or videos or website or web site*).ti.

41. (book or books or video or videos or website or web site*).ab. /freq=2

42. or/9-10,39-41

43. 38 and 42

44. 37 or 43

# PsycINFO

1. mass media/ or films/ or exp news media/ or exp printed communications media/ or animation/ or public service announcements/

2. digital video/ or audiovisual communications media/ or videotapes/

3. internet/ or exp social media/ or exp telecommunications media/ or websites/

4. music/ or rock music/

5. or/1-4

6. exp health education/

7. health promotion/

8. 6 or 7

9. 5 and 8

10. (storybook* or comic* or ebook* or e-book* or story or poem* or poetry or comic strip* or photonovella* or photo novella* or fotonovela* or photo diary or photo diaries or photodiary or photodiaries or flipchart* or flip chart* or storyboard* or story board* or printed health material*).mp.

11. (pamphlet* or information sheet* or newsletter* or digital animation* or cartoon* or gif or infographic* or podcast* or social media or twitter or facebook or blog or blogs or face book or song or songs or youtube).mp.

12. (book or books or video* or website or web site* or game or games or app or smart phone* or smartphone*).mp.

13. or/10-12

14. ((health or consumer* or patient* or parent* or caregiver*) adj2 (information or education*)).mp.

15. 13 and 14

16. exp health behavior/

17. exp behavior modification/

18. ((health or illness) adj behavio?r).mp.

19. (self adj (manag* or care or administ* or monitor* or efficac* or medicat* or mainten* or treat*)).mp.

20. or/16-19

21. 9 or 15

22. 20 and 21

23. knowledge transfer/

24. (research adj2 ("use" or utili#ation or adopt* or implement* or disseminat* or uptake or support)).mp.

25. (knowledge adj2 ("use" or utili#ation or adopt* or implement* or disseminat* or uptake or transfer* or translat* or support)).mp.

26. (evidence adj2 ("use" or utili#ation or adopt* or implement* or disseminat* or uptake or transfer* or translat* or support)).mp.

27. (implementation adj1 (science or research or intervention)).mp.

28. information dissemination/

29. evidence based practice/

30. health knowledge/ or "knowledge (general)"/

31. knowledge.ti.

32. or/23-31

33. 21 and 32

34. 22 or 33

35. or/24-27

36. 13 and 35

37. (game or games or app or smart phone* or smartphone*).mp.

38. (book or books or video* or website or web site*).ti.

39. (book or books or video* or website or web site*).ab. /freq=2

40. 37 or 38 or 39

41. 23 or 24 or 25 or 26 or 27

42. 40 and 41

43. 34 or 42

44. limit 43 to yr="2000 -Current"

# CINAHL

| S1 | (MH "Books") OR (MH "Pamphlets") OR (MH "Audiorecording") OR (MH "Communications Media") OR (MH "Multimedia") OR (MH "Videorecording+") OR (MH "Electronic Books") OR (MH "Print Materials") OR (MH "Social Media") OR (MH "Internet+") OR (MH "Music") |
| --- | --- |
| S2 | (MH "Consumer Health Information") |
| S3 | S1 AND S2 |
| S4 | ( storybook* or comic* or ebook* or "e-book*" or story or poem* or poetry ) OR ( "comic strip*" or photonovella* or "photo novella*" or fotonovela* ) OR ( "photo diary" or "photo diaries" or photodiary or photodiaries ) OR ( flipchart* or "flip chart*" or storyboard* or "story board*" or "printed health material*" ) OR ( pamphlet* or "information sheet*" or newsletter* or "digital animation*" ) OR ( cartoon* or gif or infographic* or podcast* or "social media" or twitter or facebook ) OR ( blog or blogs or face book or song or songs or youtube ) OR ( book or books or video* or website or "web site*" or game or games or app or "smart phone*" or smartphone* ) |
| S5 | ( caregiver* N0 (information or education*) ) OR ( parent* N0 (information or education*) ) OR ( patient* N0 (information or education*) ) OR ( consumer N0 (information or education*) ) OR ( health N0 (information or education*) ) |
| S6 | (MH "Consumer Health Information") |
| S7 | S5 OR S6 |
| S8 | S4 AND S7 |
| S9 | S3 OR S8 |
| S10 | (MH "Health Behavior+") OR (MH "Self Care+") OR (MH "Self Medication") OR (MH "Disease Management") |
| S11 | ( "health behavio#r" OR "illness behavio#r" ) OR ( self N0 (manag* or care or administ* or monitor* or efficac* or medicat* or mainten* or treat*) ) |
| S12 | S10 OR S11 |
| S13 | S9 AND S12 |
| S14 | (MH "Diffusion of Innovation") OR (MH "Professional Practice, Evidence-Based+") OR (MH "Knowledge+") OR (MH "Health Knowledge") |
| S15 | research N2 ("use" or utili#ation or adopt* or implement* or disseminat* or uptake or support) |
| S16 | knowledge N2 ("use" or utili#ation or adopt* or implement* or disseminat* or uptake or transfer* or translat* or support) |
| S17 | evidence N2 ("use" or utili#ation or adopt* or implement* or disseminat* or uptake or transfer* or translat* or support) |
| S18 | (implementation N1 (science or research or intervention)) |
| S19 | S14 OR S15 OR S16 OR S17 OR S18 |
| S20 | S9 AND S19 |
| S21 | S13 OR S20 |
| S22 | TI evidence N2 ("use" or utili#ation or adopt* or implement* or disseminat* or uptake or transfer* or translat* or support) |
| S23 | TI knowledge N2 ("use" or utili#ation or adopt* or implement* or disseminat* or uptake or transfer* or translat* or support) |
| S24 | TI research N2 ("use" or utili#ation or adopt* or implement* or disseminat* or uptake or support) |
| S25 | S22 OR S23 OR S24 |
| S26 | S18 OR S25 |
| S27 | S4 AND S26 |
| S28 | S21 OR S27 |
| S29 | S21 OR S27  Limiters - Published Date: 20000101-20161231; Research Article |

# SocINDEX

| S1 | ( storybook* or comic* or ebook* or "e-book*" or story or poem* or poetry ) OR ( "comic strip*" or photonovella* or "photo novella*" or fotonovela* ) OR ( "photo diary" or "photo diaries" or photodiary or photodiaries ) OR ( flipchart* or "flip chart*" or storyboard* or "story board*" or "printed health material*" ) OR ( pamphlet* or "information sheet*" or newsletter* or "digital animation*" ) OR ( cartoon* or gif or infographic* or podcast* or "social media" or twitter or facebook ) OR ( blog or blogs or face book or song or songs or youtube ) OR ( book or books or video* or website or "web site*" or game or games or app or "smart phone*" or smartphone* ) |
| --- | --- |
| S2 | ( caregiver* N0 (information or education*) ) OR ( parent* N0 (information or education*) ) OR ( patient* N0 (information or education*) ) OR ( consumer N0 (information or education*) ) OR ( health N0 (information or education*) ) |
| S3 | S1 AND S2 |
| S4 | ( "health behavio#r" OR "illness behavio#r" ) OR ( self N0 (manag* or care or administ* or monitor* or efficac* or medicat* or mainten* or treat*) ) |
| S5 | S3 AND S4 |
| S6 | research N2 ("use" or utili#ation or adopt* or implement* or disseminat* or uptake or support) |
| S7 | knowledge N2 ("use" or utili#ation or adopt* or implement* or disseminat* or uptake or transfer* or translat* or support) |
| S8 | evidence N2 ("use" or utili#ation or adopt* or implement* or disseminat* or uptake or transfer* or translat* or support) |
| S9 | (implementation N1 (science or research or intervention)) |
| S10 | S6 OR S7 OR S8 OR S9 |
| S11 | S3 AND S10 |
| S12 | S5 OR S11 |
| S13 | S5 OR S11 |
| S14 | TI ( storybook* or comic* or ebook* or "e-book*" or story or poem* or poetry ) OR ( "comic strip*" or photonovella* or "photo novella*" or fotonovela* ) OR ( "photo diary" or "photo diaries" or photodiary or photodiaries ) OR ( flipchart* or "flip chart*" or storyboard* or "story board*" or "printed health material*" ) OR ( pamphlet* or "information sheet*" or newsletter* or "digital animation*" ) OR ( cartoon* or gif or infographic* or podcast* or "social media" or twitter or facebook ) OR ( blog or blogs or face book or song or songs or youtube ) OR ( book or books or video* or website or "web site*" or game or games or app or "smart phone*" or smartphone* ) |
| S15 | S10 AND S14 |
| S16 | S13 OR S15 |
| S17 | S13 OR S15  Limiters - Date of Publication: 20000101-20151231 |

# Web of Science: Science Citation Index Expanded 1900-; Social Sciences Citation Index 1900-; Conference Proceedings Citation Index- Science 1990-; Conference Proceedings Citation Index- Social Science & Humanities 1990-; Book Citation Index– Science 2005-; Book Citation Index– Social Sciences & Humanities 2005-

| #1 | TS=( storybook* or cartoon* or comic* or ebook* or "e-book*" or story or poem* or poetry ) OR TS=( "comic strip*" or photonovella* or "photo novella*" or fotonovela* ) OR TS=("photo diary" or "photo diaries" or photodiary or photodiaries ) OR TS=( flipchart* or "flip chart*" or storyboard* or "story board*" or "printed health material*" ) OR TS=( pamphlet* or "information sheet*" or newsletter* or "digital animation*" ) OR TS=( cartoon* or gif or infographic* or podcast* or "social media" or twitter or facebook ) OR TS=( blog or blogs or "face book" or song or songs or youtube ) OR TS=( book or books or video* or website or "web site*" or game or games or app or "smart phone*" or smartphone* )  Indexes=SCI-EXPANDED, SSCI, CPCI-S, CPCI-SSH, BKCI-S, BKCI-SSH Timespan=2000-2015 |
| --- | --- |
| #2 | TS=("caregiver* information" or "caregiver* education*" OR "parent* information" or "parent* education*" OR "patient* information" or "patient* education*" OR "consumer information" or "consumer education*" OR "health information" or "health education*")  Indexes=SCI-EXPANDED, SSCI, CPCI-S, CPCI-SSH, BKCI-S, BKCI-SSH Timespan=2000-2015 |
| #3 | TS=("health behavior" OR "health behaviour" or "illness behavior" or "illness behaviour" or "self manag*" or "self care" or "self administ*" or "self monitor*" or "self efficac*" or "self medicat*" or "self mainten*" or "self treat*")  Indexes=SCI-EXPANDED, SSCI, CPCI-S, CPCI-SSH, BKCI-S, BKCI-SSH Timespan=2000-2015 |
| #4 | #1 AND #2 AND #3 |
| #5 | TS=("research use" or "research utilisation" or "research utilization" or "research adopt*" or "research implement*" or "research disseminat*" or "research uptake" or "research support")  Indexes=SCI-EXPANDED, SSCI, CPCI-S, CPCI-SSH, BKCI-S, BKCI-SSH Timespan=2000-2015 |
| #6 | TS=("knowledge use" or "knowledge utilisation" or "knowledge utilization" or "knowledge adopt*" or "knowledge implement*" or "knowledge disseminat*" or "knowledge uptake" or "knowledge support" or "knowledge transfer" or "knowledge translation")  Indexes=SCI-EXPANDED, SSCI, CPCI-S, CPCI-SSH, BKCI-S, BKCI-SSH Timespan=2000-2015 |
| #7 | TS=("evidence use" or "evidence utilisation" or "evidence utilization" or "evidence adopt*" or "evidence implement*" or "evidence disseminat*" or "evidence uptake" or "evidence support" or "evidence transfer" or "evidence translation")  Indexes=SCI-EXPANDED, SSCI, CPCI-S, CPCI-SSH, BKCI-S, BKCI-SSH Timespan=2000-2015 |
| #8 | TS=("implementation science" or "implementation research" or "implementation intervention")  Indexes=SCI-EXPANDED, SSCI, CPCI-S, CPCI-SSH, BKCI-S, BKCI-SSH Timespan=2000-2015 |
| #9 | #5 OR #6 OR #7 OR #8 |
| #10 | #1 AND #9 |
| #11 | #4 OR #10 |
