# Supplementary material for: Knowledge translation tools for parents on child health topics: a scoping review
Source: BMC Health Serv Res. 2017 Sep 29;17:686. doi: 10.1186/s12913-017-2632-2 (PMC5622461; doi:10.1186/s12913-017-2632-2)
Supplement: Supplementary file 2 — Secondary screening criteria. (DOCX 17 kb) [file 12913_2017_2632_MOESM2_ESM.docx]

**Additional file 2: Secondary screening criteria**

Guiding Screening Question: **Does the study evaluate the effectiveness of a knowledge translation tool for health consumers?**

**Yes = include**

**No = exclude**

To answer “Yes” to the above guiding question, the study must meet the following parameters:

**1. Is effectiveness evaluated?**

The focus of effectiveness evaluation is ‘did this work’ not accessibility, usability, feasibility, whether it was used/accessed.

Yes = move to next question

Unsure = move to next question

No = exclude study

**2. Is the target audience a recipient of healthcare and/or a participant in the health decision-making process for/with a healthcare recipient?**

Other audiences (i.e., clinicians) can be included, but the healthcare consumer intervention must be described and outcome measures and results reported separately.

Yes = move to next question

Unsure = move to next question

No = exclude study

3. **Is the intervention a stand-alone (i.e., not embedded in another program/KT intervention), user-mediated (i.e., on-demand) product designed to put synthesized health research knowledge into practice?**

Can be included if it is one-arm of a multi-arm study. Examples include: website, video, pamphlet, booklet, etc. An intervention is not user-mediated if the product is only viewed 1 time within the control of the research team (i.e., in a hospital/clinic, in a lab) immediately followed by post-intervention evaluation measures.

Yes = include study

Unsure = mark study as unsure

No = exclude study
