# Supplementary material for: Knowledge translation tools for parents on child health topics: a scoping review
Source: BMC Health Serv Res. 2017 Sep 29;17:686. doi: 10.1186/s12913-017-2632-2 (PMC5622461; doi:10.1186/s12913-017-2632-2)
Supplement: Supplementary file 3 — WIDER recommendations to improve reporting of the content of behavior change interventions. (DOCX 14 kb) [file 12913_2017_2632_MOESM3_ESM.docx]

**Additional file 3:** **WIDER recommendations to improve reporting of the content of behavior change interventions**

| **WIDER Recommendations** | **Supplementary Recommendations** |
| --- | --- |
| Detailed description of interventions in published papers | 1) characteristics of those delivering the intervention  2) characteristics of the recipients  3) the setting  4) the mode of delivery  5) the intensity  6) the duration  7) adherence/fidelity to delivery protocols  8) detailed description of the intervention content provided for each study group |
| Clarification of assumed change process and design principles | 1) the intervention development  2) the change techniques used in the intervention  3) the causal processes targeted by these change techniques |
| Access to intervention manuals/protocols, | Submit protocols or manuals for publication to make these supplementary materials easily accessible (*i.e.*, online). |
| Detailed description of active control conditions | 1) control intervention described  2) control intervention not described  3) no active control (i.e., standard care/no intervention control)  4) no control group |
