# Supplementary material for: Knowledge translation tools for parents on child health topics: a scoping review
Source: BMC Health Serv Res. 2017 Sep 29;17:686. doi: 10.1186/s12913-017-2632-2 (PMC5622461; doi:10.1186/s12913-017-2632-2)
Supplement: Supplementary file 4 — Outcomes of interest for assessing patient-focused interventions. (DOCX 15 kb) [file 12913_2017_2632_MOESM4_ESM.docx]

**Additional file 4:** **Outcomes of interest for assessing patient-focused interventions**

| **Outcome category** | **Examples** |
| --- | --- |
| Patients’ knowledge | - Knowledge of condition and long term complications - Self-care knowledge - Knowledge of treatment options and likely outcomes - Comprehension of information - Recall of information |
| Patients’ experience | - Patient satisfaction - Doctor-patient communication - Quality of life - Psychological wellbeing - Self-efficacy - Patient involvement |
| Service utilization and costs | - Hospital admissions - Emergency admissions - Length of hospital stay - GP visits - Cost-effectiveness - Cost to patients - Days lost from work/school |
| Health behaviour and health status | - Self-care activities - Treatment adherence - Disease severity/activity - Symptom control - Functional ability - Clinical indicators |
