# Supplementary material for: Knowledge translation tools for parents on child health topics: a scoping review
Source: BMC Health Serv Res. 2017 Sep 29;17:686. doi: 10.1186/s12913-017-2632-2 (PMC5622461; doi:10.1186/s12913-017-2632-2)
Supplement: Supplementary file 5 — Summary of included studies (n=18). (DOCX 32 kb) [file 12913_2017_2632_MOESM5_ESM.docx]

**Additional file 5: Summary of Included Studies (n=18)**

| **Author**  **(Year)**  **Country** | **Study Focus**  **(child health topic)** | **Study Population (sample size)** | **Intervention & Comparison Groups** | **Primary outcome(s) category(ies)**  **(specific outcome)** | **Results by outcome category** | **Author Conclusions** |
| --- | --- | --- | --- | --- | --- | --- |
| **Cross-sectional studies (n=4)** | | | | | | |
| Dempsey et al. (2006)  USA | Public health (Vaccination) | Parents of children 8-12 years old (n=1600 parents) | **KT^[[1]](#footnote-1)^ tool intervention**: Information sheet | Patients’ experience (vaccine acceptability) | No effect | "Providing parents with a written information sheet about HPV did lead to improvement in their knowledge about HPV but did not result in substantial increases in HPV vaccine acceptability" (p. 1492). |
|  |  |  | **Comparison**: No intervention control |  |  |  |
| Evans et al. (2009)  United Kingdom | Chronic  (Inherited metabolic disorders) | Children on low protein diet & their caregivers (n=102 patients) | **KT tool intervention:** Video + book | Health behaviour/health status (self-reported change in frequency of low protein cooking) | **Unclear:** Descriptive statistics provided for child & caregiver on related outcome (willingness to try new recipe); self-reported change in frequency of low protein cooking not reported. | "The book and/or DVD did not engage families who chose not to routinely cook with low protein ingredients" (p. 412). |
|  |  |  | **Comparison:** None |  |  |  |
| Ranjit et al. (2015)  USA | Public health (Healthy diet & physical activity) | Parents (n=322 parents) | **KT tool intervention**: book (bilingual) | Patients’ knowledge (healthy eating, physical activity) | No effect | "A narrative communication approach presented as a book of role model stories can bring about a positive change in lifestyle behaviours and associated cognitions" (p. 99). |
|  |  |  |  | Patients’ experience (self-efficacy, perceived barriers related to healthy eating & physical activity) | **Mixed effect:** Significant +ve effect on self-efficacy measures, but no effect on perceived barrier measures. |  |
|  |  |  | **Comparison**: Participants who did not read book | Health behaviour/health status (intentions related to healthy eating & physical activity) | No effect |  |
| Sustersic et al. (2013)  France | Acute (Gastroenteritis, tonsillitis) | Adult parents of pediatric patients^[[2]](#footnote-2)^ (n=154 pediatric patients) | **KT tool intervention**: Pamphlet | Health behaviour/health status (related to pediatric tonsillitis or gastroenteritis) | **Mixed effect:** No effect for child health groups (n=2) regardless of condition. Significant +ve effect for childhood gastroenteritis group compared to control, no effect for childhood tonsillitis group compared to control.^[[3]](#footnote-3)^ | "The four PIL studies significantly improved patient knowledge and increased patient autonomy by inducing behaviour closer to that recommended by the guidelines" (p. 30-31). |
|  |  |  | **Comparison**: Usual care control |  |  |  |
| **Before-after studies (n=1)** | | | | | | |
| Skranes et al. (2015)  Norway | Public health  (Child health, minor conditions) | Mothers of young children (n=99 mothers) | **KT tool intervention**: website | Patients’ knowledge (child health) | Significant +ve effect | "Regular use of a website about child health developed by experienced physicians enhanced perceived parents competence, reduced anxiety and increased knowledge among Norwegian mothers of young children" (p. 83) |
|  |  |  | **Comparison**: Same participants before viewing website | Patients’ experience (self-perceived anxiety) | No effect |  |
| **Controlled before-after studies (n=2)** | | | | | | |
| Scheinman et al. (2010)  USA | Public health  (Infant feeding) | Latina mothers of children ≤ 24 months old (n=439 women) | **KT tool intervention**: Video (bilingual) | Patients’ knowledge (age-appropriate infant feeding practices) | **Mixed effect:** Significant +ve effects on 3/9 measures at 6 months; no effect on 6/9 measures at 6 months. | "We found that an inexpensive, low-intensity video intervention can positively impact maternal knowledge and behaviour related to infant feeding among Latinas" (p. 464). |
|  |  |  | **Comparison**: No intervention control | Health behaviour/health status (actual infant feeding practices) | **Mixed effect:** Significant +ve effects on 1/7 measures at 6 months; no effect on 6/7 measures at 6 months. |  |
| Taddio et al. (2014)  Canada | Acute (Procedural pain management) | New mothers in hospital following birth of child (n=440 mothers) | **KT tool intervention**: Pamphlet | Patients’ knowledge (vaccination pain management) | No effect | "This study did not support passive dissemination of the pamphlet in hospital postnatal discharge packages as a method of educating new parents about pain management during infant vaccinations" (p. 81). |
|  |  |  | **Comparison:** Pre-intervention group |  |  |  |
|  |  |  | **Comparison**: No intervention control |  |  |  |
| **Cohort study (n=1)** | | | | | | |
| Nordfeldt (2002)  Sweden | Chronic  (Type I diabetes) | Children with type I diabetes & their caregivers (n=122-139 patients from 1994-1999) | **KT tool intervention**: 2 pamphlets (hypoglycaemia & tools) + 2 videos (hypoglycaemia & tools) with patient/parent Q&A | Patients’ experience (perceived benefit) | **Unclear:** Significant +ve effect for 1 video (hypoglycaemia) vs 1 brochure (hypoglycaemia). Overall effect of brochure vs videos + brochures or tools brochure vs tools video not reported. | "Targeted self-study material for home use that supports diabetes self-care and aims at the prevention of severe hypoglycaemia may be used as a complement to regular visits to the diabetes team" (p. 136). |
|  |  |  | **KT tool comparison**: 2 pamphlets (hypoglycaemia & tools) | Health behaviour/health status (episodes of severe hypoglycaemia, HbA1c level) | **Unclear:** No effect for brochure group only on average incidence of severe hypoglycaemia. No results reported for video + brochure group. Significant decrease in HbA1c for brochure group only. No results reported for video + brochure group. |  |
| **Randomized controlled trial (n=10)** | | | | | | |
| Bailey et al. (2015)  Australia | Acute (Surgery pain management) | Parents of children undergoing surgery (n=58 patients) | **KT tool intervention**: Information sheet | Patients’ knowledge (pain control) | Significant +ve effect | "The primary objective to explore the efficacy of the information sheet has proved to be successful in this setting. Thus, an information sheet included in the parent and patient shared decision model of analgesia leads to improved control in the management of postoperative analgesia" (p. 736). |
|  |  |  |  | Patients’ experience (satisfaction with post-surgery pain control) | Significant +ve effect |  |
|  |  |  | **Comparison**: Usual care control | Health behaviour/health status (pain as rated separately by child and parent) | **Mixed effect:** Significant +ve effect on pain measures at 2/3 time points (parent measure) and 1/3 time points (child measure). |  |
| Bauchner et al. (2001)  USA | Public health (Antibiotics use) | Parents of children 6 months - 3 years old (n=206 parents) | **KT tool intervention**: Video +pamphlet | Patients’ knowledge (appropriate use of antibiotics, reasons for development of bacterial resistance) | No effect | "Overall this video had only a modest effect on parent knowledge, beliefs, and self-reported behaviours regarding oral antibiotics. We believe that any campaign promoting the judicious use of oral antibiotics must use a multifaceted approach and target both parents and physicians" (p.845). |
|  |  |  | **Comparison**: No intervention control | Patients’ experience (beliefs about antibiotics & bacterial resistance) | No effect |  |
|  |  |  |  | Health behaviour/health status (administration and use of antibiotics) | **Mixed effect:** Significant +ve adjusted difference for 1/5 behaviour items |  |
| Christakis et al. (2006)  USA | Public health (Preventive care for common childhood conditions) | Parents of children <11 years old attending health clinics for well-child visits (n=887 families) | **Intervention (not KT tool):** Tailored website + HCP notification | Health behaviour/health status (discussion with HCP, implementation of prevention practices) | **Mixed effect:** Significant +ve effect for all interventions combined (n=3) compared to control on behaviour measures. Website + notification group and notification alone group had significant +ve effects on discussion with HCP measures compared to control, but no effect for website-alone group compared to control. Website + notification group and website-alone group had significant +ve effects on implementation measures compared to control, but no effect for notification-alone group compared to control. | "A web-based intervention can activate parents to discuss prevention topics with their child's provider. Delivery of tailored content can promote preventive practices" (p. 1157). |
|  |  |  | **KT tool intervention**: Tailored website |  |  |  |
|  |  |  | **Intervention (not KT tool):** HCP notification |  |  |  |
|  |  |  | **Comparison**: Usual care control |  |  |  |
| Jackson et al. (2006)  USA | Public health (Smoking prevention) | Smoking parents & their non-smoking children attending grade 3 (n=776 children) | **KT tool intervention**: Printed activity guide (n=5) + series of tip sheets for parents + series of newsletters for children | Health behaviour/health status (initiation of smoking) | Significant +ve effect | "Children in the pre-initiation phase of smoking who are exposed to antismoking socialization from their parents are less likely to try smoking, even if their parents smoke" (p. 61). |
|  |  |  | **KT tool comparison**: Information sheet (n=5) |  |  |  |
| Nordfeldt et al. (2003)  Sweden | Chronic  (Type I diabetes) | Caregivers of children < 19 years with Type I diabetes (n=332 patients) | **KT tool intervention**: 2 video + booklet re: skills for self-control & treatment | Health behaviour/health status (yearly incidence of severe hypoglycemia needing assistance, Hba1c level – at 1 year) | **Mixed effect**: Significant +ve effect for intervention group on yearly incidence of hypoglycaemia measure. No effect for traditional or control group. No effect for all groups on yearly mean HbA1c level measure. | "We found that a pedagogical device for home use that supports diabetes self-care and is especially targeted at the prevention of severe hypoglycaemia may contribute to a decrease in severe hypoglycaemia without worsened metabolic control" (p. 244). |
|  |  |  | **KT tool comparison**: 1 video + booklet re: general diabetes info |  |  |  |
|  |  |  | **Comparison**: Usual care control |  |  |  |
| Nordfeldt et al. (2005)  Sweden | Chronic  (Type I diabetes) | Caregivers of children < 19 years with Type I diabetes (n=332 patients) | **KT tool intervention**: 2 videos + booklet re: skills for self-control & treatment | Health behaviour/health status (yearly incidence of severe hypoglycemia needing assistance – at 2 years) | Significant +ve effect | "We found that a self-study material for home use that supports diabetes self-care targeted at the prevention of severe hypoglycaemia may contribute to a decrease in severe hypoglycaemia" (p. 1400). |
|  |  |  | **KT tool comparison**: 1 video + booklet re: general diabetes info |  |  |  |
|  |  |  | **Comparison**: Usual care control |  |  |  |
| Reich et al. (2010)  USA | Public health  (Child health, minor conditions) | Pregnant women (n=198) | **KT tool intervention**: Books (n=6) | Patients’ knowledge (anticipatory guidance topics regarding children from birth to 12 months) | **Mixed effect**: No group-time effect. Significant +ve effect for intervention vs both comparison, no effect between comparison groups (pairwise, time removed from model). | "We found that embedding anticipatory guidance into baby books is an effective was to increase new mothers' knowledge of injury prevention and healthy development in the first year" (p. 1001). |
|  |  |  | **Comparison**: Non-educational books (n=6) |  |  |  |
|  |  |  | **Comparison**: No intervention control |  |  |  |
| Tijam et al. (2013)  The Netherlands | Chronic  (Vision impairment) | Parents of children 3-6 years old with low SES^[[4]](#footnote-4)^ (n=114 children) | **KT tool intervention**: Cartoon book | Health behaviour/health status (compliance with occlusion therapy) | **Mixed effect:** Significant +ve effect for all interventions combined compared to control. Significant +ve effect for 1/3 intervention pairs (cartoon story vs reward calendar), but not 2/3 pairs (reward calendar vs leaflet; cartoon story vs leaflet). | "While our data only shows that compliance improved significantly in children who used the self-explanatory cartoon story, but not in the control group, we believe that a similarly designed educational cartoon story could also be useful in the long-term treatment of other diseases in young children" (p. 328). |
|  |  |  | **KT tool intervention**: Pamphlet |  |  |  |
|  |  |  | **Intervention (not KT tool):** Stickers |  |  |  |
|  |  |  | **Comparison:** Colouring pictures |  |  |  |
| Wakimizu et al. (2009)  Japan | Acute  (Herniorrhaphy surgery) | Parents of children undergoing surgery (n=158) | **KT tool intervention:** take home video + booklet | Patients’ knowledge (degree of information provided to child) | **Mixed effect:** Significant +ve effect on 2/5 knowledge measures. | "Children and caregivers who watched the video together as frequently as they wanted at home in a relaxed atmosphere were better informed and prepared and they exhibited less anxiety regarding surgical hospitalization than those who watched the same video once at the outpatient clinic a week before surgery" (p.400). |
|  |  |  | **Comparison (not KT tool)**: in clinic video + booklet | Patients’ experience (child anxiety, parent anxiety) | **Mixed effect:** Significant +ve group - time effect on child anxiety measures and significant +ve effect at perioperative period (but no other periods). No effect for parent anxiety measures, but significant +ve effect at post-operative period (no other period). |  |
| Wilson et al. (2006)  USA | Public health (Vaccination) | Low income mothers (n=54 mothers) | **KT tool intervention**: Pamphlet (n=2) | Patients’ knowledge (vaccines) | No effect | "Although there was a modest increase in immunization knowledge for both groups, it was not significant. Thus, simplifying information alone may not increase parental knowledge" (p.4). |
|  |  |  | **KT tool comparison**: Information sheet (n=2) |  |  |  |

1. KT = Knowledge translation [↑](#footnote-ref-1)
2. Additional study population not included in this review was adult patients with tonsillitis and gastroenteritis. [↑](#footnote-ref-2)
3. Significant +ve effect for adult groups (n=2), adult gastroenteritis group, and adult tonsillitis group compared to control. [↑](#footnote-ref-3)
4. Socio-economic status [↑](#footnote-ref-4)
